# Effect of Ni-Based Superalloy on the Composition and Lifetime of Aluminide Coatings

**DOI:** 10.3390/ma18133138

**Published:** 2025-07-02

**Authors:** Maryana Zagula-Yavorska

**Affiliations:** Faculty of Mechanical Engineering and Aeronautics, Rzeszow University of Technology, al. Powstańców Warszawy 12, 35-959 Rzeszów, Poland; yavorska@prz.edu.pl

**Keywords:** high-temperature low-activity vapor-phase process, refractory elements, interplanar distance of the β-NiAl phase, air exposure

## Abstract

Aluminide coatings on nickel-based superalloys were synthesized via a high-temperature “clean” low-activity vapor-phase process. This process is environmentally friendly and meets manufacturers’ environmental protection requirements. Hence, it fulfils the Industry 4.0 requirements, where the reduction of environmental impact in the industrial sector is a key issue. Surface morphology, cross-section microstructure, and phase composition of the coatings were studied and compared by using an optical microscope and a scanning electron microscope (SEM) equipped with an energy dispersive spectroscope (EDS) and X-ray diffraction (XRD). Bare and coated superalloys’ lifetime was evaluated and compared via air exposure at 1100 °C. High-temperature low-activity aluminizing of the IN713, IN625, and CMSX4 superalloys enabled the obtainment of the desirable β-NiAl phase. The highest nickel content in the chemical composition of the IN713 superalloy among the investigated superalloys resulted in the highest aluminide coatings’ thickness. Moreover, the higher refractory elements concentration in the IN625 and CMSX4 superalloys than that in the IN713 superalloy may contribute to a thinner aluminide coatings’ thickness. Refractory elements diffused to the surface of the superalloy and formed carbides or intermetallic phases, which impeded outward nickel diffusion from the substrate to the surface and thereby inhibited coating growth. The obtained coatings fulfilled the requirements of ASTM B 875. Despite the fact that the coating formed on IN713 was thicker than that formed on IN625, the lifetime of both coated superalloys was comparable. Oxidation resistance of the aluminide coatings formed on the IN713 and IN625 superalloys makes them the favored choice for gas turbine applications.

## 1. Introduction

The performance of an aircraft engine turbine improves by increasing the temperature of the gases introduced into the turbine [1]. Increasing the gas temperature from 900 to 1250 °C causes a 30% increase in the output power [2]. The most critical elements of an aircraft engine turbine are turbine blades. They are subject to strong mechanical stresses and work in a highly corrosive environment [3,4,5]. Therefore, a turbine blade must exhibit the following properties: (1) resistance to thermal fatigue, creep, and mechanical fatigue; (2) resistance to hot corrosion and oxidation; (3) high microstructural stability; and (4) resistance to erosion. Ni-based superalloys have a high melting temperature, creep and fatigue resistance, therefore, hot part elements in gas turbines are made from them [6,7,8,9,10]. The necessity of protection of the hot part elements from high working temperatures and oxidation requires the use of special coating systems on their surface [11]. Thermal barrier coatings (TBCs) are multilayer coating systems consisting of an intermediate layer (*bond coat*) and a ceramic top layer (*top coat*) designed to protect the elements from oxidation and high working temperatures, respectively [12,13,14,15]. The ceramic top layer is ca. 100–400 µm thick and consists of yttrium oxide partially stabilized by zirconium oxide. Two groups of bond coatings are generally used [12,13,14,15]. The first group is overlay coatings with a typical MCrAlX composition, where M denotes Ni, Co, or Fe and X means oxygen-reactive elements, namely Zr, Si, Hf, and Y [16,17,18]. The composition of the coating is selected in such a way that the microstructure consists of a β phase in a γ matrix. Overlay coatings are deposited by thermal spray or physical vapor deposition. The adhesion of overlay coatings to the substrate produced by thermal spray or physical vapor deposition methods is limited. The aspect of adhesion is fundamental: in rotating turbine blades running at thousands of revolutions per minute, a lack of a coating will cause destruction of the engine. The second group is diffusion aluminide coatings [19,20,21,22]. The β-NiAl phase is the main phase of these coatings. Low density, high melting temperature, high elasticity modulus, and good adherence to the substrate are the distinctive features of the β-NiAl phase. These properties make the β-NiAl phase very beneficial for use in coatings. The efficiency of aluminide coatings is related to the presence of the intermetallic aluminum-rich matrix layer, which is an aluminum reservoir to form an α-Al_2_O_3_ protective scale. The strongly compact structure of the scale phase ensures a drastic reduction of the inward diffusion of oxygen ions and hence improves the oxidation performance of nickel-based superalloys above 1000 °C.

Aluminide coatings have often been deposited by means of the pack cementation method [23,24,25]. The formation of a β-NiAl coating is a result of the reaction between the mixed powder and coated elements. The formation of coatings by pack cementation has been investigated widely [23,24,25]. It was found that the composition and structure of coatings depend on the kind of the substrate [23,24,25]. This means that coatings are usually tailor-made for a specific alloy. Low cost and good coating reproducibility are the main advantages of the pack cementation method. However, particles used in this method may be entrapped in the outer part of the coating. Hence, the continuity of the coating is hindered.

Out-of-the pack aluminizing is an alternative method, in which coated components are situated above the powder mixture containing aluminum [26]. Coatings produced by the out-of-the pack aluminizing method are much cleaner and more homogeneous than those produced by pack aluminizing.

Slurry aluminizing is an alternative method used to produce oxidation-resistant coatings [27]. The slurry is a suspension created by mixing a binder with the aluminum powder. The suspension is sprayed on the superalloy substrate, dried, and then heat-treated to obtain a diffused coating. However, the heat treatment of slurry coatings leads to the formation of volatile organic compounds that contain phosphorus and chromium derivatives. Therefore, the use of slurry coatings is limited.

Vapor-phase aluminizing is a successive technological method used to produce oxidation-resistant coatings [28,29,30]. Aluminum pellets are used in this process. Simplification of the discharge and charge processes of the aluminum source is an advantage, since no volatile dust is created. Vapor-phase aluminizing becomes the preferred aluminizing method due to the excellent process control, the cleanliness of the process, the fact that there is no need for high vacuum, no reactions during the heating and cooling periods, and the homogeneity of the produced coating. Coatings on a nickel superalloy substrate obtained in the abovementioned process are used in F100 military engines, which power the F-16 and F-15 fighter aircrafts.

Vapor-phase aluminizing can usually be performed in three different ways: low-temperature high-activity—performed below 950 °C (LTHA), high-temperature high-activity (HTHA), and high-temperature low-activity—performed above 1000 °C (HTLA) [28,29,30]. The diffusion rate of aluminum is lower than that of nickel in HTLA aluminizing. Therefore, the nickel aluminide coating grows outwardly, and the β-NiAl phase is the main phase of the coating. The diffusion rate of aluminum is greater than that of nickel in LTHA aluminizing. Therefore, the nickel aluminide coating grows inwardly, and the Al-rich δ-Ni_2_Al_3_ is the main phase of the coating. The nickel diffusion rate is higher than that in LTHA aluminizing and is comparable with the aluminum diffusion rate in HTHA aluminizing. Furthermore, the initial growth of HTHA coatings is controlled by aluminum’s inward diffusion and then by nickel’s outward diffusion. As a result, the aluminide coating consists of only the β-NiAl phase or both the β-NiAl and δ-Ni_2_Al_3_ phases. The presence of the δ-Ni_2_Al_3_ phase is not preferred because of its brittleness and instability at temperatures higher than 1133 °C. The undesired δ-Ni_2_Al_3_ phase is changed into the desired β-NiAl phase by an additional heat treatment process, which is carried out at ca. 1100 °C for a few hours [31]. The aluminized coating’s microstructure consists of two layers: the interdiffusion layer and the additive layer. The interdiffusion layer forms between the additive layer and the substrate due to the compositional change between them and consists of a matrix phase with different types of precipitates depending on the kind of the substrate.

Mo et al. [32] produced aluminide coatings on a K444 nickel-based superalloy at 850, 900, 950, 1000, and 1050 °C for 1.5 h by means of the vapor-phase aluminizing method. A bilayer structure was observed. The outer zone of the coating was the β-NiAl phase, while the interdiffusion zone was mostly comprised of nickel–aluminum intermetallics, topologically close-packed phases, and carbides. Christoglou et al. [33] produced aluminide coatings on ARMCO iron at 1000 °C for 4 h by means of the fluidized bed chemical vapor deposition (FB-CVD) method. A double-layer structure was observed. The outer zone of the coating was the FeAl phase, while the interdiffusion zone was composed of a solid solution of aluminum in α-Fe. Wang et al. [34] deposited aluminide coatings on Haynes 188 and WI-52 cobalt-based superalloys at 1080 °C for 4 h by means of vapor-phase aluminizing with the use of 15Al–85Cr and 44Al–56Cr nuggets. Aluminide coatings deposited on Haynes 188 with 15Al–85Cr and 44Al–56Cr nuggets consisted of a β-(Co,Ni)Al phase outer layer and a Cr-rich + W-rich precipitated phase interdiffusion layer; on the other hand, aluminide coatings deposited on WI-52 with 15Al–85Cr nuggets consisted of a CoAl phase outer layer and a Cr-rich + W-rich precipitated phase interdiffusion layer.

The coatings used within the turbine differ according to the considered rotor stage. Blades of the first stage are coated with a platinum-modified aluminide, while turbine blades of the third stage are coated with an unmodified aluminide because of the low temperatures in which they work.

World manufacturers of aircraft engines—Pratt and Whitney, Rolls-Royce, or General Electric—look for an aluminizing process which is environmentally friendly, repeatable, and ensures the production of aluminide coatings with uniform thickness on both the external and internal surfaces of cooling channels of turbine blades.

It was found that the compositional difference between the substrate and the coatings ensures the driving force for the various elements’ interdiffusion when the coated superalloy is subjected to high temperature [7,10,11]. One of the main elements of the aluminide coating and the substrate is aluminum. The loss of aluminum from the aluminide coating toward the surface is well-understood. However, the loss of aluminum from the aluminide coating toward the substrate is not explained. A large aluminum concentration lowers the melting point of superalloys. Chromium is a constituent of superalloys. It improves the oxidation resistance of superalloys to 816 °C, decreases the aluminum requirement for alumina scale formation, and lowers creep strength. Nickel diffuses outwardly to form NiAl for a NiCr alloy with 10 wt.% chromium during low-activity aluminizing. The alloy loses nickel near the interface. On the other hand, the alloy becomes enriched with chromium near the interface. The chromium level is within the limit of solubility, and no chromium particles occur. A chromium-saturated NiAl phase forms during the low-activity aluminizing of a Ni10Cr alloy. Nickel diffuses outwardly to form NiAl for a NiCr alloy with 20 wt.% chromium during low-activity aluminizing. The alloy loses nickel and increases the chromium concentration. An increase in the chromium concentration at the near-interface region exceeds the limit of solubility and leads to α-Cr precipitates near the interface in the alloy. Precipitation of α-Cr in the coating reduces ductility and oxidation resistance of coating. Refractory elements such as molybdenum, tungsten, or rhenium diffuse to the surface of the superalloy and form carbides MC, M_6_C, M_23_C_6_ or intermetallic phases, which impede outward nickel diffusion from the substrate to the surface, thereby inhibiting the coating’s growth. The literature data indicate that the composition and structure of coatings depend on the substrate [23,24,25]. In turn, the chemical and phase composition of coatings influences the lifetime of superalloys [23,24,25]. This means that coatings are tailor-made for an individual alloy. There is a lack of information on how compositions of substrates affect the microstructure of aluminide coatings and their lifetime at high temperature. To fill these gaps, this study provides new insights regarding the analysis of the structure of aluminide coatings on the IN713, IN625, and CMSX4 Ni-based superalloys and their oxidation resistance at elevated temperatures. Superalloys were taken as the substrate since they are used in the hot parts of aircraft engines. Aluminide coatings were produced via a “clean” chemical vapor deposition process, which meets manufacturers’ environmental protection requirements, thus fulfils the Industry 4.0 requirements, where reduction of environmental impact is a key factor.

## 2. Materials and Methods

The IN713 (polycrystalline), IN625 (polycrystalline), and CMSX4 (monocrystalline) Ni-based superalloys were used as the substrate. The nominal compositions of the IN713, IN625, and CMSX4 superalloys are shown in Table 1 [6,35,36].

The cylinder-shaped superalloys with a diameter of 15 mm and a height of 4 mm were cut, ground up to SiC No1000, and degreased in ethanol. Finally, the samples were aluminized. High-temperature low-activity aluminizing (HTLA) was performed through a chemical vapor deposition process. AlCl_3_ halide (IonBond Company, Olten, Switzerland) was an aluminum source. Hydrogen as the carrier (IonBond Company, Olten, Switzerland) gas supplied the halide to the superalloy samples. The aluminizing process consisted of the following steps:

I—heating from room temperature to 1050 °C;
II—aluminizing at 1050 °C for 8 h;
III—cooling samples with a furnace to 500 °C;
IV—cooling samples in ambient air.

**Figure 1:**
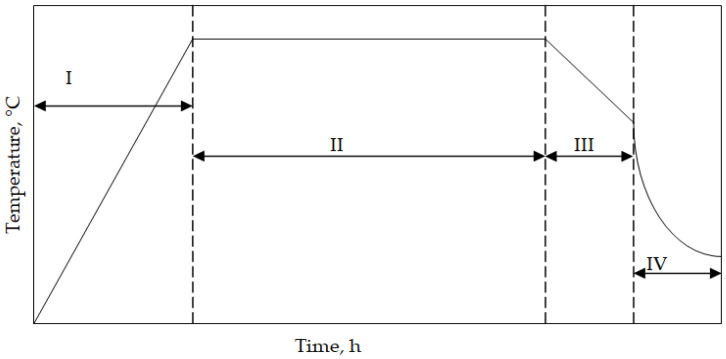
A scheme of the temperature profile of CVD aluminizing.

A temperature scheme during the chemical vapor deposition aluminizing process is shown in Figure 1. The elaborated process is “clean” and meets manufacturers’ environmental protection requirements. Hence, it fulfils the Industry 4.0 requirements, where the reduction of environmental impact is a key factor.

Aluminide coatings’ surfaces were investigated with a Hitachi S-3400N scanning electron microscope (SEM) (Hitachi America, Ltd., Iowa City, IA, USA) equipped with an energy dispersive spectroscope (EDS) (Hitachi America, Ltd., Iowa City, IA, USA). Then, coated samples were cut, mounted, gritted, and polished to get metallurgical samples. The thickness of the coatings was measured using a Nikon Epiphot 300 optical microscope (Nikon, Tokyo, Japan). A phase analysis of the coatings was performed by means of X-ray diffraction (XRD) (XTRa ARL, Waltham, MA, USA) using Cu-K_α_ radiation in the 10–100° interval. An oxidation test was carried out in a muffle furnace (Czylok, Jastrzębie-Zdrój, Poland) in the air at 1100 °C. The furnace was heated up to 1100 °C; then, the coated samples were placed there and held for 20 h. Then, the samples were taken out, air-cooled for 2 h to room temperature, and weighed with an accuracy of 0.0001 g. Uniform air flow was maintained. The above action was a cycle, while the total oxidation time was ca. 400 h. The oxidation time was that long to record the loss of lifetime of the samples.

## 3. Results and Discussion

### 3.1. Effect of a Ni-Based Superalloy on the Surface Morphology, Microstructure, Chemical and Phase Composition of Coatings

**Figure 2:**
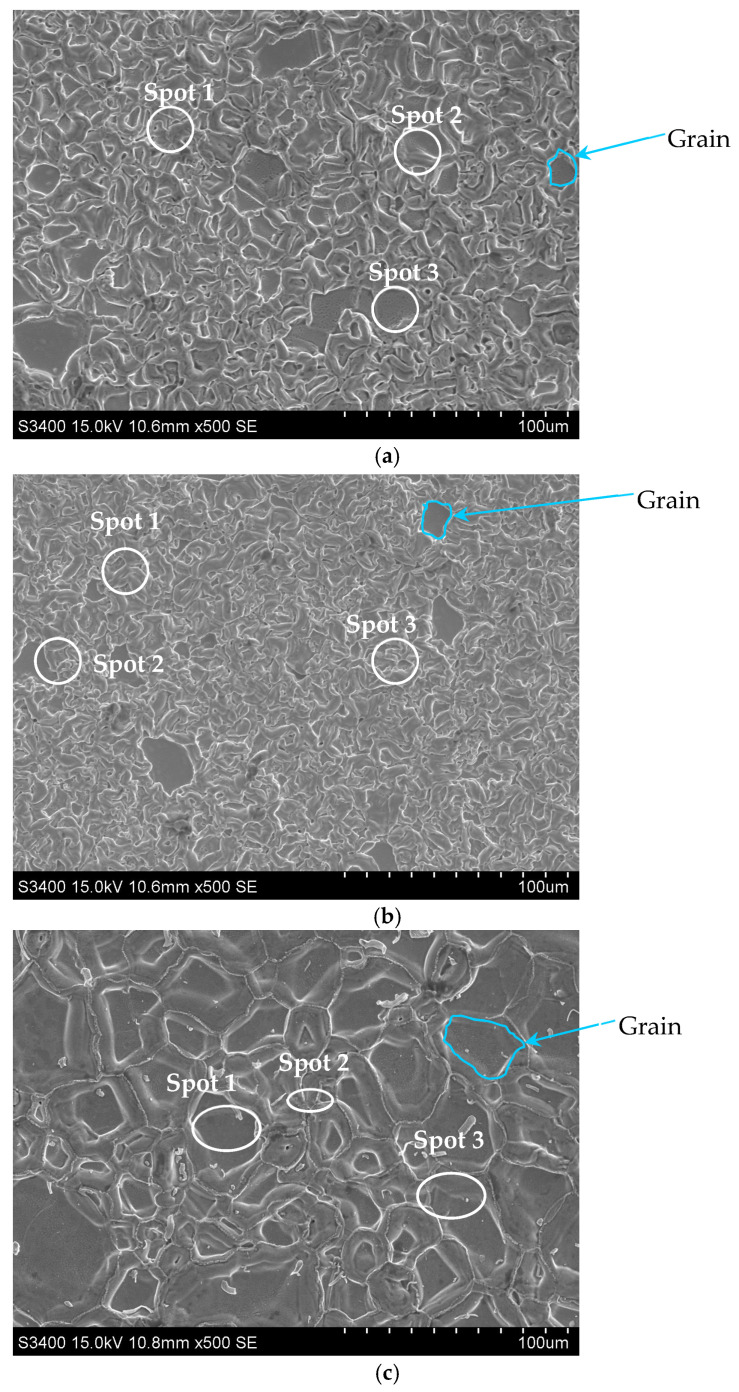
Surface morphology of the aluminide coatings deposited on the IN713 (**a**), IN625 (**b**), and CMSX4 (**c**) superalloys.

**Figure 3:**
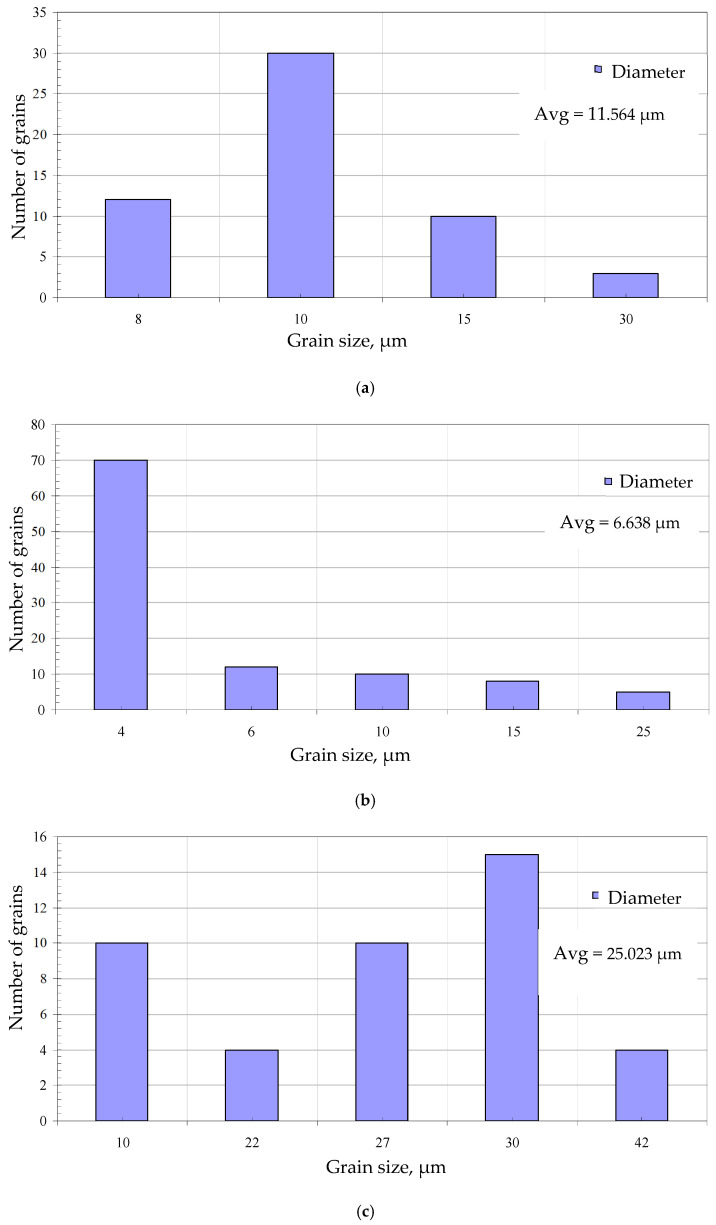
Grain size distribution histogram from the SEM images of the surfaces of the aluminide coatings deposited on the IN713 (**a**), IN625 (**b**), and CMSX4 (**c**) superalloys.

The surface morphology with EDS spectrum results of the aluminide coatings deposited on IN713, IN625, and CMSX4 is shown in Figure 2a–c and Table 2, Table 3 and Table 4. The size of the grains was determined from SEM images by using grain size histograms, as shown in Figure 3. The fine-grain structure of the coatings’ surfaces was observed. The average grain diameter on the surface of the IN713 superalloy coating was ca. 12 µm, whereas the average grain diameter on the surface of the IN625 superalloy coating was ca. 7 µm—slightly smaller than that on the IN713 superalloy coating. The average grain diameter on the surface of the CMSX4 superalloy coating was ca. 25 µm. The coating comprised mainly aluminum, nickel, and a small amount of the elements that were in the substrate: molybdenum and chromium (IN713); chromium and iron (IN625); or cobalt and chromium (CMSX4). The aluminum and nickel contents on the surface of the coatings indicated the presence of a β-NiAl phase (Table 2, Table 3 and Table 4).

**Figure 4:**
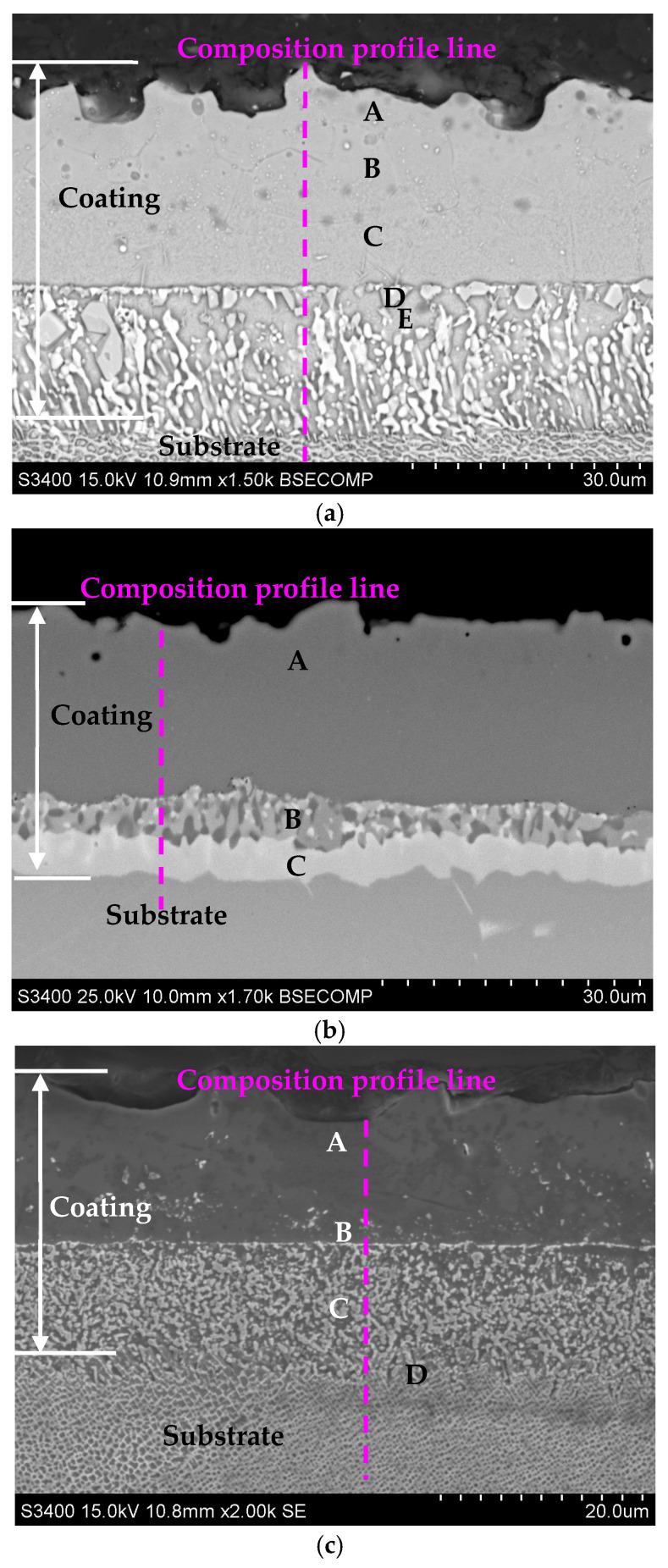
Cross-sections of the aluminide coatings deposited on the IN713 (**a**), IN625 (**b**), and CMSX4 (**c**) superalloys.

The cross-sections of the aluminide coatings deposited on IN713, IN625, and CMSX4 with EDS spectrum results are shown in Figure 4a–c and Table 5, Table 6 and Table 7. The coatings’ cross-sections consisted of two layers: outer and interdiffusion. The outer layer comprised mainly aluminum, nickel, and a small amount of the elements that were in the substrate: chromium, molybdenum, and titanium (IN713); chromium and molybdenum (IN625); or cobalt, chromium, and titanium (CMSX4). The aluminum and nickel contents in the outer layers of the coatings were within a range from 42 to 47 at.% and from 50 to 56 at.%, respectively. This indicated the presence of NiAl alloyed by the substrate elements.

The interdiffusion layer located under the outer one was composed of precipitate phases enriched with refractory elements that diffused from the substrate. High chromium concentrations (71 and 81 at.%) were identified in the interdiffusion layer of the coatings on the IN713 and IN625 substrates, respectively (Figure 4a,b, Table 5 and Table 6). Furthermore, the precipitated phases in the interdiffusion layer are, probably, Cr_23_C_6_ carbides [35], while chromium (12 at.%), tungsten (11 at.%), and rhenium (5 at.%) were mainly identified in the interdiffusion layer of the coating on the CMSX4 substrate (Figure 4c, Table 7). The precipitated phases in the interdiffusion layer are, probably, topologically close-packed (TCP) phases [36].

**Figure 5:**
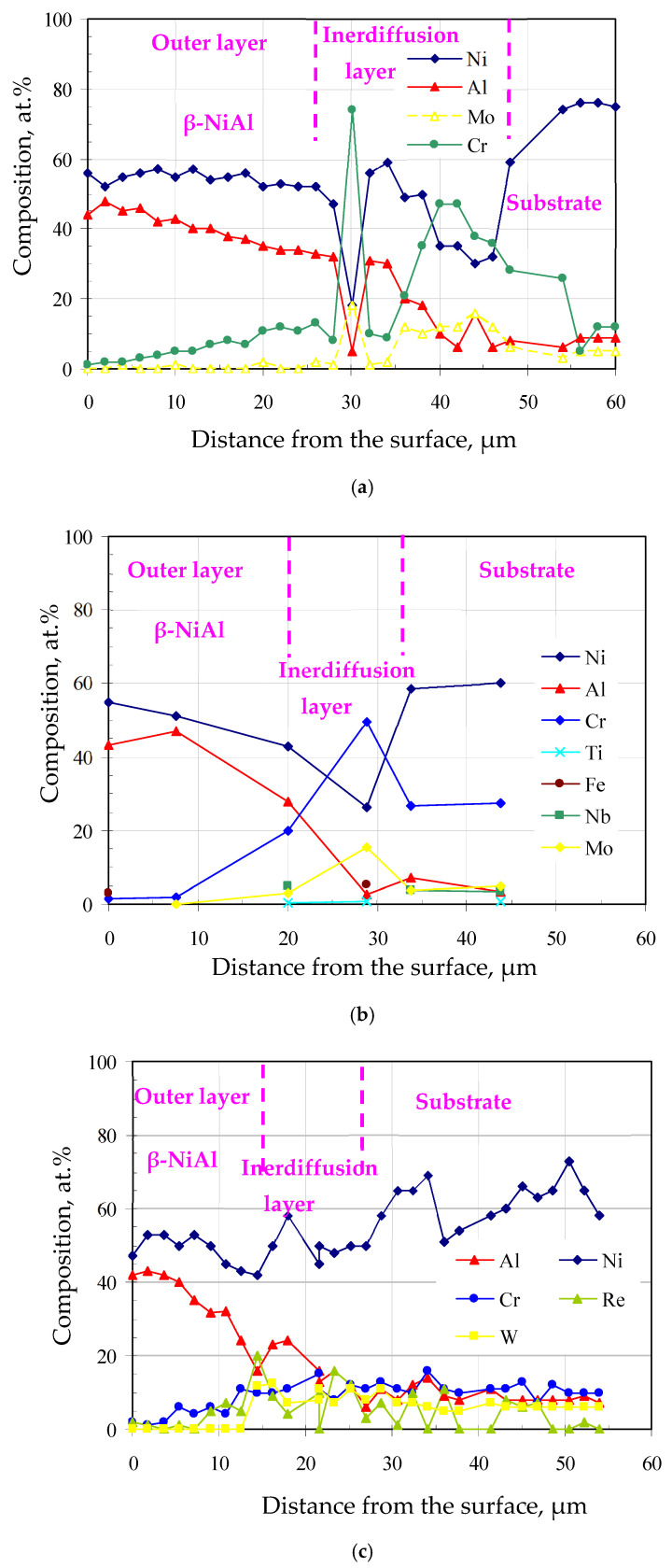
Chemical composition of the cross-sections of the aluminide coatings deposited on the IN713 (**a**), IN625 (**b**), and CMSX4 (**c**) superalloys.

The chemical composition profiles on the cross-sections of the aluminide coatings are shown in Figure 5a–c. The aluminum content decreased from the outer layer to the substrate, while the nickel content was nearly constant in the outer layer and decreased in the interdiffusion layer. Moreover, the chromium concentration in the outer layer of the coatings was much lower than that in the interdiffusion layer. This is as a result of the lower outward chromium diffusion rate in the NiAl phase formed during aluminizing [37].

**Figure 6:**
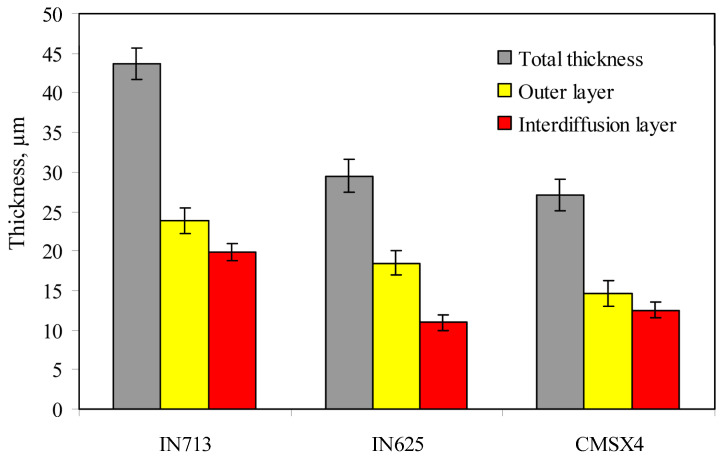
Thickness of the aluminide layers deposited on the IN713, IN625, and CMSX4 superalloys.

The thickness of the aluminide layers deposited on the IN713, IN625, and CMSX4 superalloys is shown in Figure 5. It was found, that the thickness of aluminide coatings depends on the chemical composition of the superalloy (Figure 6). The thickness of the aluminide coatings on the IN713 superalloy ranged from 41 to 46 µm (the outer layer was from 22 to 26 µm thick, while the interdiffusion layer was from 19 to 21 µm thick). The aluminide coating on the IN713 superalloy was the thickest among the investigated ones. The thickness of the aluminide coatings on the IN625 superalloy ranged from 28 to 31 µm (the outer layer was from 18 to 20 µm thick, while the interdiffusion layer was from 9 to 12 µm thick). The thickness of the aluminide coatings on the CMSX4 superalloy was similar to the thickness of the coatings on the IN625 superalloy, from 26 to 30 µm (the outer layer was from 14 to 16 µm thick, while the interdiffusion layer was from 12 to 13 µm thick). Thus, the aluminide coatings on the IN625 and CMSX4 superalloys were 14 µm thinner than those on the IN713 superalloy.

**Figure 7:**
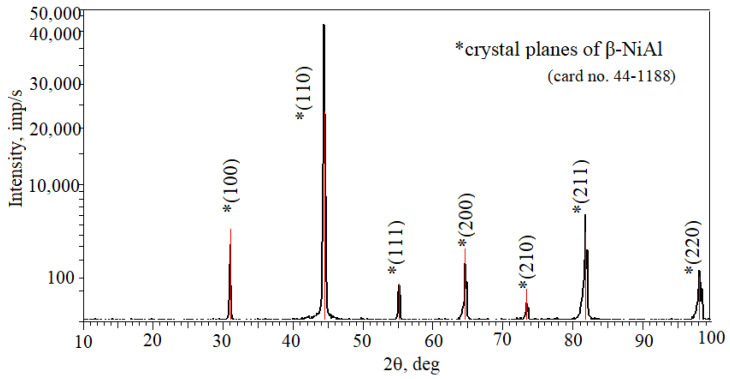
X-ray diffraction results of the coating surface of the IN713 superalloy.

X-ray diffraction results of the coating surfaces are shown in Figure 7. All reflections correspond to the interplanar distance of the β-NiAl phase.

The highest nickel content in the IN713 superalloy (74.2 wt.%) among all the investigated superalloys resulted in the highest aluminide coating thickness. The nickel content in the IN625 and CMSX4 superalloys is the same (62 wt.%). It may result in a similar aluminide coating thickness (30 µm). Moreover, the higher concentration of refractory elements in the IN625 and CMSX4 superalloys than that in the IN713 superalloy may contribute to a thinner aluminide coating thickness. Refractory elements diffuse to the surface of the superalloy and form carbides MC, M_6_C, M_23_C_6_ or intermetallic phases, which impede outward nickel diffusion from the substrate to the surface, thereby inhibiting coating growth.

Aluminide coatings’ formation can be considered a one-dimensional semi-infinite process that takes place according to Fick’s second law [14]:

(1) $$\frac{\partial \emptyset }{\partial t}=D\frac{\partial^{2}\emptyset }{\partial x^{2}}$$

where Ø is the mass flux, t is the time, D is the diffusion coefficient, and x denotes the distance.

A qualitative mechanism of the diffusion aluminide formation on nickel superalloys was developed by Goward and Bone [14]. It was found that diffusivities of nickel and aluminum depend on the stoichiometry of NiAl. *D*_Ni_/*D*_Al_ is ca. 3 for a nickel-rich NiAl phase with an aluminum content less than 50 at.%. *D*_Ni_/*D*_Al_ is more than 3 for an aluminum-rich NiAl phase with an aluminum content of ca. 51.5 at.%. The aluminum activity plays a main role in predicting the predominant diffusing species. Nickel diffusion predominates in a hypostoichiometric composition (aluminum content less than 50 at.%). The coating comprises two layers, an interdiffusion layer (IL) and an outer layer (OL). The outer layer is comprised of the AlNi phase saturated with the elements of the substrate. The formation mechanism of coatings can be described by the primarily outward nickel diffusion from the substrate. Nickel that diffused outwardly from the substrate reacts with aluminum from the halides to form an AlNi compound [37]. The area below the initial surface is depleted of nickel due to its outward diffusion. Precipitation of carbides or intermetallic phases was caused by a decrease in nickel content in this area [37,38].

According to ASTM B 875, the minimum aluminide coating thickness on nickel-based alloys has to be 25 µm, and the outer 15% of the coating shall contain a minimum of 41 at.% aluminum [39]. The aluminide coatings’ thickness ranged from 26 to 46 µm, and the aluminum content in the coating ranged from 42 to 47 at.% on the IN713, IN625, and CMSX4 nickel superalloys. Thus, the obtained coatings fulfilled the requirements of ASTM B 875.

### 3.2. Effect of Ni-Based Superalloys on the Lifetime of Coatings

**Figure 8:**
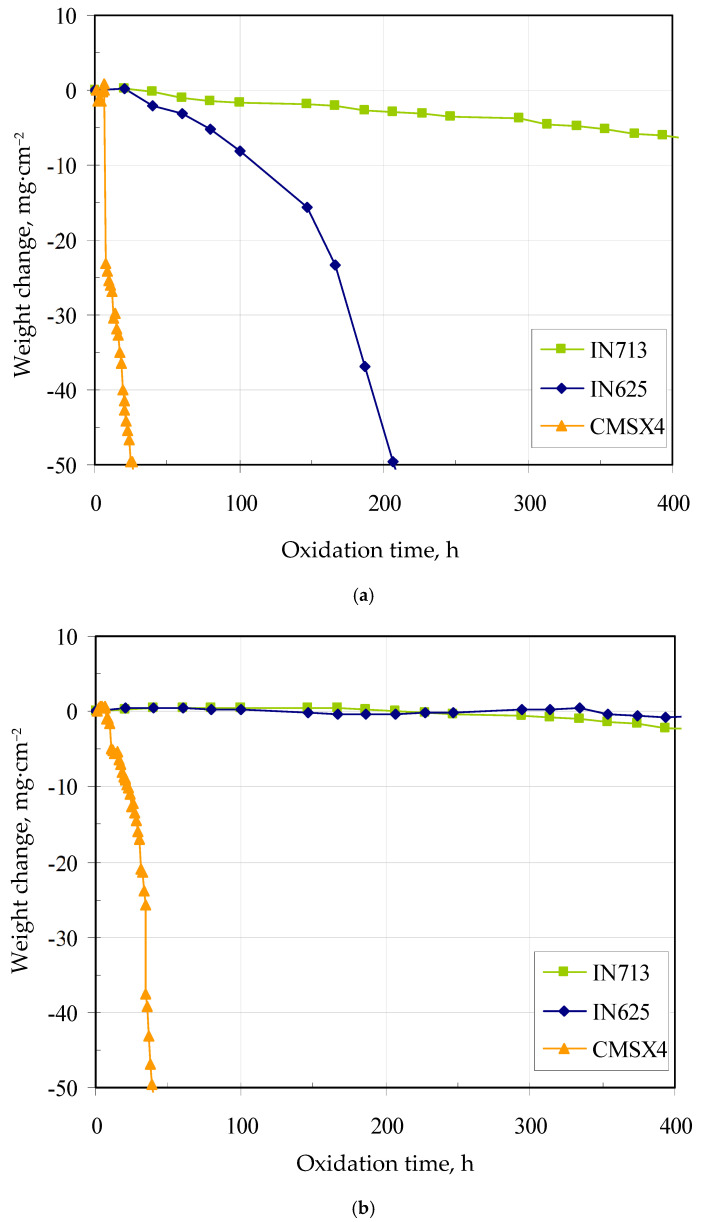
Weight change curves of the bare (**a**) and coated (**b**) superalloys.

Weight change curves of the bare and coated superalloys are shown in Figure 8a,b. The weight change of bare superalloys decreases just after 40 h of oxidation. This is due to the scarce protective properties of the scales formed on their surface. The worst lifetime and rapid weight loss were found for the bare CMSX4 superalloy. Similar oxidation kinetics were observed for the PWA 1480 single-crystal superalloy with 8–10 ppm of sulfur [40]. Sulfur is present in superalloys as an impurity in raw materials and at each step of the superalloy processing [40]. During oxidation, sulfur segregates at the oxide/superalloy interface and accelerates the growth of voids and cracks, which results in a reduction of adhesion of the oxide scale to the superalloy [40,41]. Smialek et al. [42] found that reducing the content of sulfur from 10 to 1 ppm in a single-crystal superalloy can greatly improve its cyclic oxidation resistance. The removal of sulfur may be achieved by repeated oxidation and polishing, high-temperature annealing in vacuum or desulphurization in hydrogen at high temperature. The removal of sulfur improves the oxidation resistance of the superalloy [40]. CMSX4 has the highest titanium content among the investigated superalloys. According to Meier et al. [43], the addition of 1 wt.% titanium improves the oxidation resistance of FeCrAl alloys. However, the addition of a similar titanium amount to nickel superalloys does not have a comparable effect. Titanium increases the growth rate of Al_2_O_3_ and has a detrimental effect on scale adhesion on nickel-based superalloys. On the other hand, Younes et al. [44] described the oxidation behavior of the CMSX4 superalloy at 1100 °C in the air up to 100 h. The scale consisted of two layers: upper and inner. The upper layer consisted of spinel (Ni,Co)Al_2_O_4_ and (Ni,Cr)_2_O_4_, the inner layer—of α-Al_2_O_3_. The lifetime of IN625 was higher than that of CMSX4 but lower than that of the IN713 superalloy. Rolland et al. [45] oxidized the IN625 superalloy at 1100 °C in the air. The oxidation rate and scale composition were evaluated. The oxides were composed of an outer chromia scale and an internal CrNbO_4_ subscale. Moreover, some oxide spallation was observed, probably due to molybdenum oxidation, leading to MoO_3_ scale evaporation and void formation at the internal interface. The best lifetime and slow weight loss were found for the bare IN713 superalloy. Lee [46] performed oxidation of the IN713 superalloy at 1100 °C in the air up to 100 h. Excellent oxidation resistance was observed. Only a few micrometer-thick scale was observed after oxidation. The scale consisted mainly of α-Al_2_O_3_, with a small amount of TiO_2_, NiAl_2_O_4_, and Cr_2_O_3._

Aluminide coatings formed via high-temperature gas-phase aluminizing increases the lifetime of the IN713, IN625, and CMSX4 superalloys. During high-temperature exposure of coated superalloys in an oxidizing environment, aluminum diffuses within the coating toward the coating surface to form or refill the protective alumina scale. Depletion of aluminum due to migration to the coating surface and interdiffusion of aluminum and nickel between the coating and the alloy substrate follow the general sequence of reactions: Ni-rich β-NiAl converts to β-NiAl + γ′; next, β-NiAl + γ′ converts to γ′ + γ; finally, γ′ + γ converts to γ [40]. The worst lifetime and rapid weight loss were found for the coated CMSX4 superalloy. It may be due to the presence of sulfur. During oxidation, sulfur may segregate at the oxide/coating interface and accelerate the growth of voids and cracks, which results in a reduction of adhesion of the oxide scale to the coated superalloy. Moreover, titanium that diffuses from the superalloy to the coating may increase the growth rate of Al_2_O_3_ and lose scale adhesion to the coated superalloy. The β-NiAl phase with a small amount of chromium (ca. 2 at.%) in the aluminide coating on the IN713 and IN625 superalloys is a reservoir of aluminum to form an α-Al_2_O_3_ protective scale. The scale phase ensures a reduction of the inward diffusion of oxygen ions, and therefore improves the oxidation performance of coated nickel-based superalloys. Despite the fact that the coatings formed on IN713 were thicker than those formed on IN625, the lifetime of both coated superalloys was comparable. Oxidation resistance of the aluminide coatings formed on the IN713 and IN625 superalloys makes them the favored choice for gas turbine applications.

## 4. Conclusions

High-temperature low-activity aluminizing of IN713, IN625, and CMSX4 enabled the obtainment of the desirable β-NiAl phase in coatings. The highest nickel content in the IN713 superalloy (74 wt.%) among the investigated superalloys resulted in the highest aluminide coatings’ thickness (41–46 µm). The concentration of Mo and Nb in IN625 is 6.4 wt.% higher than that in IN713, resulting in a 30% reduction in coating thickness. Refractory elements diffused to the surface of the superalloy and formed carbides or intermetallic phases, which impeded outward nickel diffusion from the substrate to the surface and inhibited coating growth. The obtained coatings fulfilled the requirements of ASTM B 875. Despite the fact that the coating formed on IN713 was thicker than that formed on IN625, the lifetime of both coated superalloys was comparable. Oxidation resistance of the aluminide coatings formed on the IN713 (41–46 µm thick, 42 at.% aluminum in the coating) and IN625 superalloys (26–31 µm thick, 47 at.% aluminum in the coating) makes them the favored choice for gas turbine applications.

## Figures and Tables

**Table 1:** Nominal chemical compositions of the IN713, IN625, and CMSX4 superalloys [6,35,36].

Element, wt.%	IN713	IN625	CMSX4
Ni	74.2	61.8	61.7
Cr	12.5	21.5	6.5
Co	-	1	9
Mo	4.2	9.0	0.6
Nb	2.0	3.6	-
Al	6.1	0.2	5.6
Ti	0.8	0.2	1
Ta	-	-	6.5
W	-	-	6
Fe	-	2.5	-
Si	0.003	0.2	-
Hf	-	-	0.1
Zr	0.1	-	-
Re	-	-	3
B	0.012	-	-
C	0.05	0.05	-

**Table 2:** Chemical composition of the surface of the aluminide coating deposited on the IN713 superalloy, at.% (Figure 2a).

Spot	Al	Ni	Cr	Mo
1	42.5	56.1	1.2	0.2
2	47.5	51.4	0.9	0.2
3	44.1	54.6	1.2	0.1
Entire analyzed area	44.7	54.0	1.1	0.2

**Table 3:** Chemical composition of the surface of the aluminide coating deposited on the IN625 superalloy, at.% (Figure 2b).

Spot	Al	Ni	Cr	Fe
1	40.7	54.8	1.4	3.1
2	36.5	59.3	1.1	3.1
3	31.1	64.1	1.6	3.2
Entire analyzed area	36.1	59.4	1.4	3.1

**Table 4:** Chemical composition of the surface of the aluminide coating deposited on the CMSX4 superalloy, at.% (Figure 2c).

Spot	Al	Ni	Cr	Co
1	44.4	49.7	1.1	4.8
2	30.9	62.9	0.7	5.5
3	45.4	48.1	0.9	5.6
Entire analyzed area	40.2	53.6	0.9	5.3

**Table 5:** Chemical composition of the cross-section of the aluminide coating deposited on the IN713 superalloy, at.% (Figure 4a).

Spot	Al	Ni	Ti	Cr	Mo
A	42.3	55.5	-	2.2	-
B	37.8	56.3	0.4	5.5	-
C	34.0	55.3	0.4	9.9	0.4
D	6.3	14.2	-	71.2	8.4
E	28.8	56.8	0.9	13.5	-
Substrate	9.0	72.4	1.0	12.3	5.3

**Table 6:** Chemical composition of the cross-section of the aluminide coating deposited on the IN625 superalloy, at.% (Figure 4b).

Spot	Al	Ni	Ti	Cr	Mo	Fe	Nb
A	47.4	50.7	-	1.8	0.1	-	-
B	1.2	7.1	-	81.2	10.5	-	-
C	2.7	26.3	1.0	49.5	15.5	5.0	-
Substrate	3.2	60.2	0.6	27.6	4.0	1	3.4

**Table 7:** Chemical composition of the cross-section of the aluminide coating deposited on the CMSX4 superalloy, at.% (Figure 4c).

Spot	Al	Ni	Ti	Cr	Mo	Co	W	Re
A	43.2	50.6	-	1.2	-	5.1	-	-
B	36.5	51.1	1.0	3.6	-	7.8	-	-
C	15.8	41.0	1.1	12.2	1.4	12.3	11.1	5.0
D	12.1	58.1	2.5	8.4	-	12.5	3.4	3.1
Substrate	7.2	58.0	2.0	10	1.0	12.0	7.0	2.8

## Data Availability

The original contributions presented in this study are included in the article. Further inquiries can be directed to the corresponding author.
